# Technical success, procedural safety, and efficacy of the Silk Vista Baby in the treatment of cerebral aneurysms over a mid-to-long-term follow-up

**DOI:** 10.3389/fneur.2024.1369443

**Published:** 2024-03-27

**Authors:** Claudio Rodríguez-Fernández, Pedro Ruiz-Garcia, Maria Jesús Garcia-Sanchez, Martiel Manrique-Zegarra, Carlos Toledano-Illán, Jorge Escartin, Miguel Angel Vences, Luis Angel Rubio, Alex Luttich, José Manuel Pumar

**Affiliations:** ^1^Interventional Neuroradiology Department, Hospital Universitario Fundación Jiménez Diaz, Madrid, Spain; ^2^School of Medicine, Universidad César Vallejo, Piura, Peru; ^3^Interventional Neuroradiology Department, Hospital Universitario Donostia, San Sebastian, Spain; ^4^Chair of Interventional Neuroradiology, University of Santiago de Compostela, Santiago de Compostela, Spain

**Keywords:** intracranial aneurysm, embolization, therapeutic, endovascular aneurysm repair, Silk Vista Baby

## Abstract

**Background:**

Long-term follow-up of cerebral aneurysms treated with the Silk Vista Baby (SVB) flow diverter is lacking. This study aimed to assess the technical success, procedural safety, and efficacy of the SVB (Balt, Montmorency, France) for the treatment of intracranial aneurysms in small cerebral vessels over a mid-to long-term follow-up.

**Methods:**

We retrospectively analyzed a prospectively maintained database of patients treated with the SVB between September 2018 and June 2021. Data regarding patient demographics, aneurysm characteristics, and technical procedures were also collected. Angiographic and clinical findings were recorded during the procedure and over a period of at least 12 months.

**Results:**

Angiographic and clinical follow-up data were available for 50 patients/50 aneurysms. The procedural complication rate was 8%. At 12 months, the final results showed a technical success rate of 100%, the re rupture rate was 0%, neuromorbidity and mortality rates of 4 and 0%, respectively, and an almost complete occlusion rate of 94%.

**Conclusion:**

Treatment of complex intracranial aneurysms with the SVB was safe and effective. Long-term results showed high rates of adequate and stable occlusions.

## 1 Introduction

Over the last decade, flow diverters (FDs) have become the endovascular treatment of choice for many intracranial aneurysms. After initial use as a last-resort strategy for giant and wide-necked aneurysms, their indications have expanded rapidly and currently include even the first treatment of incidental saccular and fusiform aneurysms located in any of the proximal segments of the circle of Willis, as well as ruptured aneurysms that could otherwise only be treated inadequately.

Bases on the optimal results obtained in the treatment of internal carotid artery (ICA) aneurysms up to the communicating segment and initial trials to establish safety and efficacy data, FDs have also been used in the treatment of aneurysms originating in small-caliber vessels; however, their use remains controversial (1, 2), mainly because these aneurysms are largely dissecting and occur in vessels with a caliber of less than 2.5 mm. Technically, these aneurysms are challenging to treat with conventional FDs because of the small diameter of the carrier vessel, more pronounced angulation of these arteries, and the need to preserve the perforating branches or branches located in bifurcations. To reduce the problems of navigability and thromboembolic complications, and improve radial strength and radiopacity, a low-profile FD has been developed specifically for the treatment of aneurysms in arteries with a caliber of less than 3.5 mm located beyond the circle of Willis (3–8).

The Silk Vista Baby (SVB) FD stent (Balt, Montmorency, France) is the first FD that can be deployed using a 0.017″ microcatheter, permitting navigation through challenging vessel anatomy and management of previously untreatable lesions. It was designed for use in intracranial vessels, including small vessels ranging from 1.5 mm to 3.5 mm in diameter.

Although several studies on the SVB have been published (9–12), no long-term follow-up has been performed. Therefore, this study investigated the technical and clinical outcomes of using the SVB in a series of patients with aneurysms of vessels with a caliber of less than 3.5 mm, and evaluated the safety of the device in terms of intraprocedural and periprocedural complication rates and its effectiveness with a follow-up of at least 12 months.

## 2 Materials and methods

### 2.1 Ethics approval

This study was approved by the local ethics committee and conducted in compliance with national legislation and the Code of Ethics Principles for Medical Research Involving Human Subjects of the World Medical Association (Declaration of Helsinki). Informed consent was obtained from all patients before treatment and chart review.

### 2.2 Study design and population

We retrospectively reviewed a prospectively maintained database of a consecutive series of patients with intracranial aneurysms treated with the SVB between September 2018 and June 2021 at our institution. This study included patients with unruptured and ruptured aneurysms of the anterior cerebral artery complex, middle cerebral artery, and vertebral and basilar arteries who were treated with the SVB. Additionally, we classified aneurysms as complex in the following situations: those with a wide neck, multilobulated formations, branching vessels emerging from the aneurysm, those associated with arterial wall defects such as dysplasia or thrombosis, and those located distally and posing challenges for vascular navigation. A multidisciplinary team of interventional neuroradiologists, neurologists, and neurosurgeons performed patient selection for endovascular treatment. The treatment decision was based on the aneurysm’s size, location, and morphology and the patient’s clinical status. The angiographic follow-up was performed with brain magnetic resonance imaging at 3 and 6 months, and with digital subtraction angiography at 12 months. The following data were collected: patient demographics, medical history, aneurysm characteristics, clinical presentation, procedural details and complications, follow-up radiological data, and functional outcomes.

### 2.3 Silk Vista Baby

The SVB is a newer iteration of the Silk Flow Diverter (Silk, Balt) designed for the endovascular treatment of intracranial aneurysms in vessels measuring 1.5–3.5 mm in diameter. It consists of 48 drawn-filled tubing nitinol/platinum wires that do not require radiopaque markers. The device is resheathed up to 90% of its length and delivered through a 0.017″ low-profile microcatheter.

### 2.4 Antiplatelet regimen

None of the patients was pretreated with dual antiplatelet therapy because our protocol comprised intraprocedural antiplatelet therapy. Once catheterization of the vascular axis on which the aneurysm depended was performed, the administration of intravenous sodium heparin was started at a dose of 50–70 U/kg of weight with 600 mg of intravenous lysine acetylsalicylate (Inyesprin) and an infusion of tirofiban at an intravenous loading dose at an initial rate of 0.4 micrograms/kg/min for 30 min. Thereafter, this was transitioned to an infusion rate of 0.1 micrograms/kg/min for 6 to 24 h depending on the type of aneurysm, patient characteristics, and risk of systemic hemorrhage. When the patient developed oral tolerance (2 h after the intervention), the clopidogrel load was administered at a dose of 600 mg, which, after 24 h, was continued at a dose of 75 mg/day with 100 mg of acetylsalicylic acid. After 6 months, clopidogrel was withdrawn (except in patients with intimal hyperplasia greater than 70%) and 100 mg of acetylsalicylic acid was maintained indefinitely.

### 2.5 Endovascular procedure

All procedures were performed with the patient under general anesthesia using an angiography system (Philips Azurion 7, Best, Netherlands) by a senior interventional neuroradiologist with extensive experience in the use of FDs.

Through a right femoral artery approach using a short 6-French introducer, the cervical segment of the ICA or the vertebral artery was accessed with a 6-F Neuron MAX 0.88-inch guide catheter (Penumbra Inc., Almeda, CA, United States) or Ballast (Balt). Once access to the vascular axis was ensured, microcatheterization of the target vessel was performed using the Headway 17 (MicroVention) or Gama 17 (Balt) microcatheters. In specific cases where navigation was more difficult owing to the distal location or complex vascular anatomy, an Excelsior SL 10 (0.0165) was used, pre-dilated with a Catch Mini 3 × 15 mm (Balt). The SVB stent deployment strategy followed strict compliance with the recommendations of the SVB device manufacturer (Balt).

### 2.6 Safety and efficacy evaluation

The primary safety endpoint was the proportion of patients who experienced death or a major stroke [>4 points higher on the National Institutes of Health Stroke Scale (NIHSS)] within 30 days after the procedure or had a major ipsilateral stroke or death within 12 months of the procedure. Neuromorbidity was defined as any neurological worsening during clinical follow-up, associated with the procedure. Patient assessments, including modified Rankin scale (mRS) and NIHSS scores, were performed at baseline and hospital discharge, and again within defined follow-up windows at 3, 6, and 12 months.

The primary efficacy endpoint was the proportion of patients with aneurysm occlusion and ≤50% stenosis of the parent artery in the intracranial aneurysm 12 months after treatment. Aneurysm occlusion was evaluated using the O’Kelly–Marotta (OKM) scale (13). The imaging was reviewed and compared by two senior endovascular interventional neuroradiologists who were not involved in the procedure for initial and follow-up occlusion grades.

### 2.7 Statistical analysis

Differences in variable distribution between groups were compared using the Kruskal–Wallis test for continuous variables and categorical data were summarized using rates and percentages. All statistical analyses were performed using SPSS software (version 22.0; IBM Corp., Armonk, New York, United States).

## 3 Results

### 3.1 Patient and aneurysm characteristics

Fifty patients (40 females and 10 males) with 50 aneurysms were included. The mean age of the included patients was 59.5 ± 12.7 years (range, 25–78 years). Thirty-five patients (70%) had unruptured aneurysms diagnosed incidentally, and 15 (30%) had ruptured aneurysms (5 treated in the acute phase and 10 had remnants after previous endovascular coil occlusion of the target aneurysm). The initial clinical status was scored as mRS scores 2, 1, and 0 in 3 (3%), 3 (3%), and 44 (88%) patients, respectively.

Overall, 72% of the aneurysms had saccular morphology, 20% were complex, and 4% were fusiform. Sixteen percent of aneurysms were located in the posterior territory. The mean size of the 50 aneurysms was 3.5 ± 2.4 mm (median, 3 mm; range, 1–17 mm), and the mean neck width was 2.7 ± 1.3 mm (median, 2 mm; range, 1–7 mm) with a neck/dome ratio of 1.3 ± 0.4 (median, 1.2; range 0.5–2.5). Overall, 20% were classified as complex aneurysms.

**Figure 1:**
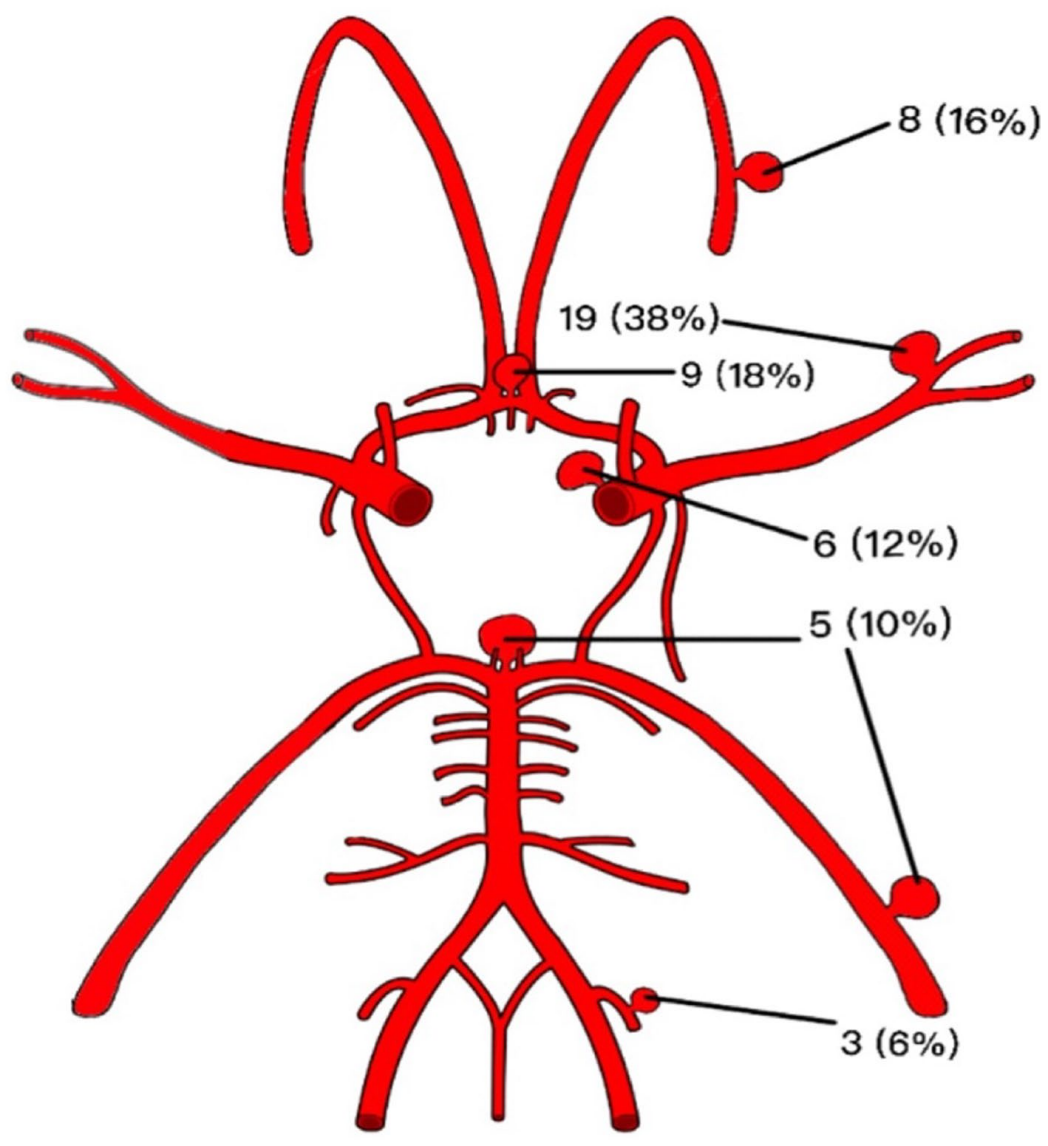
Graphic summary of the anatomical distribution and frequencies corresponding to the treated aneurysmal lesions.

Of the 50 aneurysms treated, 6 (12%) were in the intracranial bifurcation of internal carotid artery, including the proximal A1 and M1 segment, 9 (18%) in the A1 and A2 portion of the artery anterior cerebral artery, 8 (16%) in the pericallosal artery, 19 (38%) in the middle cerebral artery, 3 (6%) in the posterior inferior cerebellar artery and 5 (10%) in the complex of the distal basilar and posterior cerebral arteries (Figure 1).

### 3.2 Endovascular procedure results

Elective treatments were conducted for 50 aneurysms across 50 interventions. Successful deployment was achieved in all but two cases, and the implantation of a second device was required because of excessive foreshortening of the first endoprosthesis. Additional coils were used for 12 aneurysms (24%), mainly in ruptured aneurysms in which an early thrombosis of the aneurysm sac was desirable, in aneurysms larger than 7 mm and in those in which the presence of a branch originating at the level of the sac or aneurysmal neck suggested thrombosis might be hindered. Balloon remodeling was performed in five cases (10%) owing to non-ideal apposition to the wall.

### 3.3 Procedural complications

**Figure 2:**
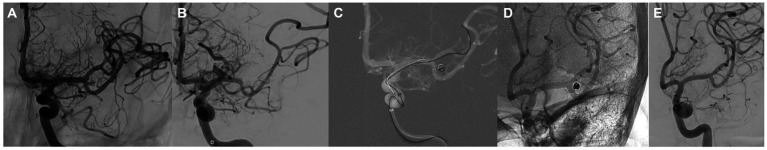
**(A)** Angiography showing an aneurysm of the bifurcation of the middle cerebral artery partially occluded by coiling. **(B)** post-SVB implantation control showing obstruction of the upper branch of the bifurcation. **(C)** Angiographic control after implantation of an Atlas stent showing reopening of the artery. **(D)** Angiographic control at 12 months showed complete occlusion of the aneurysm and patency of the upper branch of M2.

Intraprocedural complications occurred in four cases (8%), all of them in non-ruptured aneurysms. One patient (2%) presented with parenchymal hemorrhage with an mRS score of 2 at the 12 months follow-up visit. In one case (2%), acute branch occlusion of the middle cerebral artery was resolved by angioplasty and implantation of an Atlas stent with an mRS score of 1 at 12 months follow-up (Figure 2). A patient (2%) presented with vasospasm, resolved by local pharmacological treatment (vasodilators and glycoprotein IIB-IIIA inhibitors), without clinical repercussions (12-month follow-up mRs score, 0). One patient (2%) presented with fluctuating motor aphasia with negative neuroimaging findings and spontaneous resolution without neurological repercussions during follow-up. The final neuromorbidity at 12 months follow-up was 4%.

### 3.4 Clinical follow-up

In the group of unruptured aneurysms (the mRS score before treatment was 0 in all cases), 1 patient developed a delayed remote hemorrhage shortly after the procedure. The 12-month mRs score was 2.

**Figure 3:**
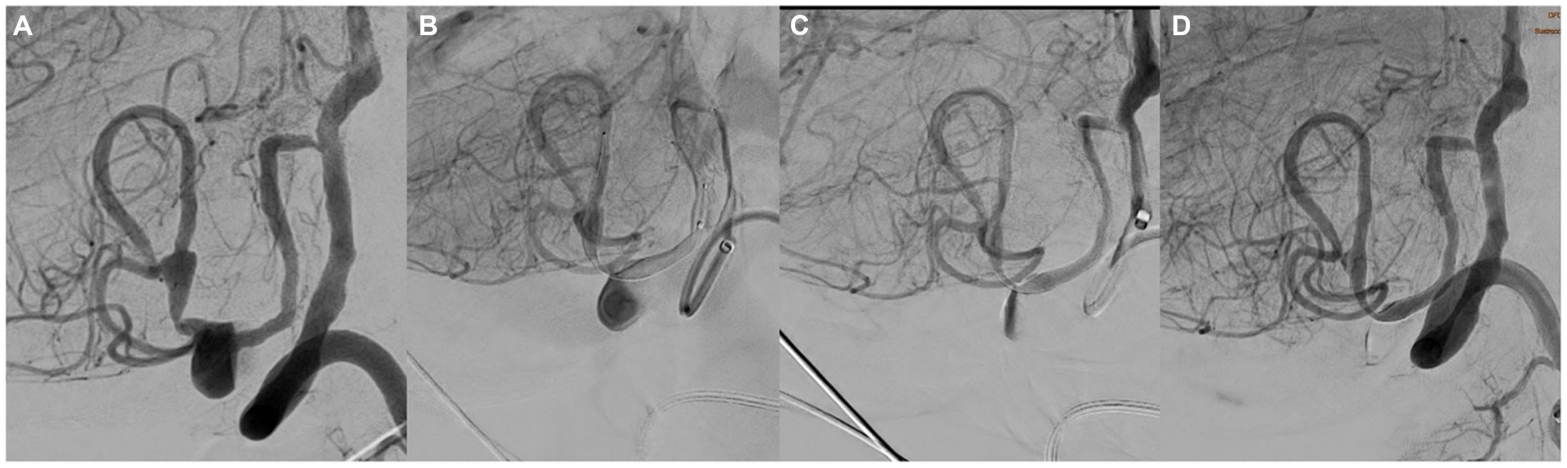
**(A)** Lateral angiography showing a dissecting aneurysm of the PICA. **(B)** Image showing the implantation of two 3.5 × 25 mm SVBs. **(C)** Angiographic control at 12 months showed complete occlusion of the aneurysm.

In the group with ruptured aneurysms, none of them suffered a re-rupture. Two patients presented with worsening of the mRs at 6 and 12 months, associated with the evolution of the disease. One patient presented with Fisher grade IV and World Federation of Neurological Surgeons (WFNS) grade V subarachnoid hemorrhage (SAH) secondary to dissection of the posterior inferior cerebellar artery. Treatment was performed on the same day as the SAH, an external ventricular drain placement and subsequent implantation of two 2.25 × 15 SVB devices. In the deferred angiographic controls performed, both devices remained patent, and complete occlusion was observed (OKM D) at 3 months, which was maintained over time. Final MRs score at 12 months follow-up was 1 point (Figure 3).

The second patient presented with Fisher grade IV and WFNS grade V SAH, secondary to the rupture of a 35 × 28-mm partially thrombosed dissecting aneurysm located in the P2 segment of the left posterior cerebral artery. The treatment included of implanting two overlapping SVBs (2.75 × 20 and 2.75 × 15) and coiling. The degree of occlusion achieved according to the OKM scale was D. The territory of the affect posterior cerebral artery showed radiological signs of chronic hemorrhagic lesion on the first follow-up controls, and the stent was observed to be occluded. No acute ischemic lesions were appreciated on magnetic resonance image. The patient’s final MRs score at 12 months was 4 points, attributed to the sequelae resulting from subarachnoid hemorrhage rather than stent occlusion.

### 3.5 Angiographic follow-up

**Figure 4:**
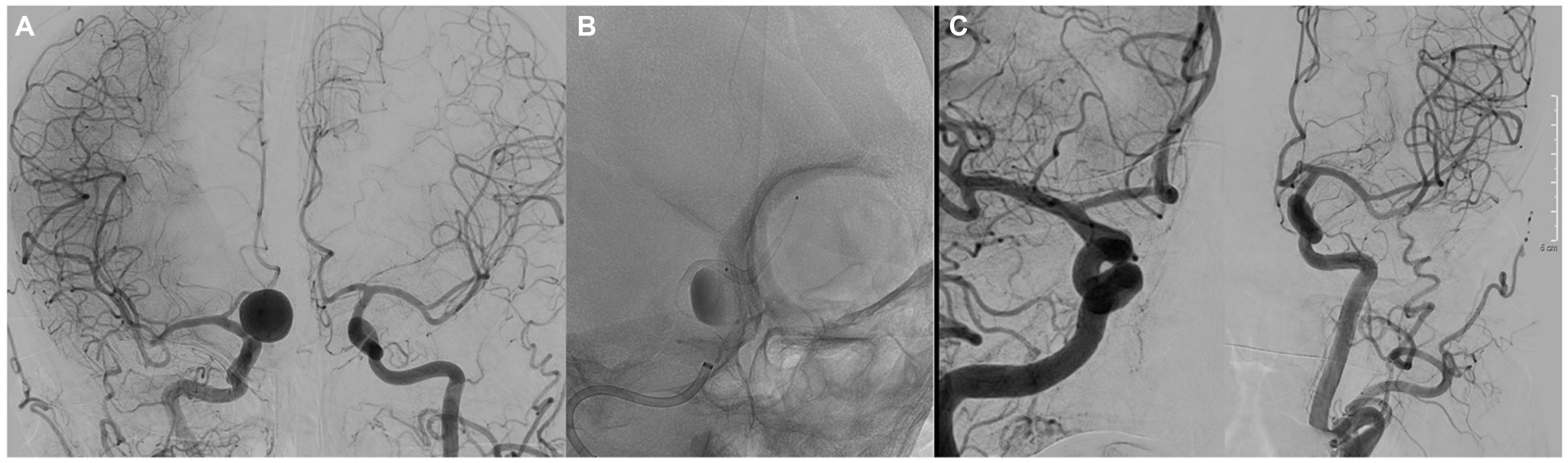
**(A)** Anteroposterior angiography showing a saccular aneurysm of the right A1 artery. **(B)** Image showing the implantation of a 3.5 × 25 mm SVB. **(C)** Angiographic control at 12 months showed complete occlusion of the aneurysm.

The angiographic follow-up was performed with brain magnetic resonance imaging at 3 and 6 months (MRA/Contrast enhanced MRA), and digital subtraction angiography at 12 months. Angiographic follow-up data were available for 50 patients, harboring a total of 50 aneurysms. In early angiographic follow-up (at 3 months),16/50 (32%) aneurysms showed complete occlusion (OKM D), and 12/50 (24%) showed near-complete aneurysm occlusion (OKM C). At 6 months, 23/50 (46.9%) (OKM D) and 17/50 (OKM C) aneurysms showed complete occlusion and near-complete aneurysm occlusion, respectively. At the 12 months follow-up, 33/50 (66%) and 14/50 (28%) aneurysms showed complete occlusion (OKM D) and near-complete aneurysm occlusion (OKM C), respectively (Figure 4). In summary, adequate aneurysmal occlusion was observed in 94% of all aneurysms after 12 months, and only 2% of the treated aneurysms remained morphologically unaltered without an apparent change in perfusion (OKM A).

Asymptomatic intimal hyperplasia inside the stent was observed at the 6 months follow-up in seven patients (14%), in whom high dose of statins was administered. In the angiographic control at 12 months, the resolution of the hyperplasia was confirmed in all patients.

## 4 Discussion

This study represents the most extensive clinical experience with the SVB based on treating of 50 patients with 50 intracranial aneurysms located in small-caliber arteries (less than 3.5 mm), with clinical angiographic follow-up at 12 months.

The potential of using FDs to treat aneurysms distal to the terminal ICA was explored before low-profile devices became available (13–16). One systematic review and meta-analysis analyzed 26 studies with 572 aneurysms, with a complete occlusion rate of 70% and procedure-related morbidity and mortality rates of 20 and 9%, respectively (17). Another meta-analysis included 129 studies with 1,654 aneurysms, with a complete occlusion rate of 76% and procedure-related morbidity and mortality rates of 5 and 4%, respectively (18). However, the results of these systematic reviews were limited by selection and publication biases of the included studies. The feasibility and high efficacy of FDs were reported for treating of aneurysms in small or distal vessels but with a non-negligible complication rate. These studies have shown promising results with a low complication rate, which means, as Brouillard et al. (19) and Patel et al. (20) reported, a new paradigm is needed to expand the indications of these devices beyond the circle of Willis.

To reduce the problem of navigability and thromboembolic complications and improve radial strength and radiopacity, three low-profile FDs have been specifically developed to treat aneurysms in arteries with calibers of less than 3.5 mm located beyond the circle of Willis (6–8): the FRED Jr. (Microvention, Tustin, CA), the p48MW flow-diverting device (Phenox GmbH, Bochum, Germany), and the SVB. The FRED Jr. and the p48MW are deployed through a 0.021-inch inner diameter microcatheter, while the SVB uses a 0.017-inch microcatheter. This specific feature of the SVB makes easier navigability, even in tortuous anatomy. In our experience with the SVB, we were able to reach the target vessel in every case.

### 4.1 Technical success of the SVB

Regarding technical complications in the deployment of low-profile FDs, Bhogal et al. (6) reported a single complication in a series of 25 patients with 25 aneurysms treated with P48, and AlMatter et al. (21) reported three complications in a series of 74 patients with 77 aneurysms. Möhlenbruch et al. (22), Rautio et al. (23), and Sivasankar et al. (5) did not observe any technical complications with the deployment of FRED Jr. The rate of intraprocedural complications observed in our series was within the expected range compared with these studies.

In our study, implantation of the SVB, even in peripheral vessels such as the M2–M3 segment, was performed without difficulty in all cases. The degree of technical success was similar to that obtained in a previously published series on the SVB (Table 1) (9–12). Consequently, delivery through a 0.017-inch microcatheter represents a significant technical advancement, allowing comparatively easy access to complicated segments of the intracranial arteries.

**Table 1:** Characteristic of the procedure and results.

Authors	No. of patients/aneurysms	Previous treatment	Complementary coiling	Additional stent	Angioplasty	Shortening	Technical success
Bhogal et al. (9)	60/61	40.9% (25/61)	21.3% (13/61)	6.6% (4/61)	0.0% (0/60)	1.6% (1/61)	98.3% (59/60)
Gavrilovic et al. (10)	18/22	Coiling: 13.6% (3/22) Clipping: 4.5% (1/22)	Not reported	9.1% (2/22)	0.0% (0/18)	0.0% (0/22)	100.0% (18/18)
Martínez-Galdámez et al. (11)	41/43	Coiling: 13.6% (5/43)	30.2% (13/43)	12.2% (6/43)	2.4% (1/41)	0.0% (0/43)	100.0% (41/41)
Schüngel et al. (12)	44/47	Coiling: 46.8% (22/47)	2.1% (1/47)	6.4% (3/47)	0.0% (0/44)	0.6% (5/47)	97.8% (44/45)
Hanel et al. (24)	163/173	32.9% (57/173)	17.9% (27/151)	8.7% (15/173)	0.6% (1/163)	2.3% (4/173)	98.8% (164/166)
Our series	50/50	Clipping: 2% (1/50) Coiling: 18% (9/50)	24% (12/50)	6% (3/50)	10% (5/50)	4% (2/50)	100% (50/50)

No., number.

### 4.2 Safety

The rates of neuromorbidity at 12-months and mortality observed in the ruptured and unruptured aneurysms treated with the SVB were 4.0 and 0.0%, respectively.

Regarding neurological morbidity and mortality, our study showed similar results than those described in the literature, which evaluated unruptured aneurysms, primarily located proximally in the ICA and treated with other FD devices. In the Pipeline for Uncoilable or Failed Aneurysms (PUFS) trial (25), the rate of acute ischemic infarction in the first 180 days of treatment was 6.5%, with a major morbidity rate of 4.7%, neurological mortality rate of 2.8%, and mortality rate of 3.7%. Brinjikji et al. (26) reported an acute ischemic event rate of approximately 5%, major morbidity rate of 6.1%, neurological mortality rate of 3.8%, and total mortality rate of 4.2%. In their prospective multicenter study of unruptured aneurysms treated with Pipeline Embolization Device (PED), Kallmes et al. (27) reported a rate of acute ischemic infarction of 4.7%, neurological morbidity rate of 6.8%, neurological mortality rate of 1.6%, and total mortality rate of 3.7%. In the PEDESTRIAN study, Lylyk et al. (28) included 835 patients treated with PED, with an ischemic infarction rate of 3.6%, major morbidity rate of 2.7%, neurological mortality rate of 3.1%, and total mortality rate of 4.6%.

In a multicenter series of 41 consecutive patients with 43 aneurysms, Martínez-Galdámez et al. (11) reported an intraoperative complication rate of 12.2%, with no clinical sequelae or changes in the mRS score compared with the patient’s baseline status at admission. Bhogal et al. (9) reported a clinical complication rate of 6.8%, with permanent morbidity (mRS score 1) in one patient (1.6%). Gavrilovic et al. (10) reported an adverse event rate of 9.1%, related to one case of branch occlusion (1/18, 5.6%) and another case of stent thrombosis (1/18, 5.6%), with an mRS score of 0 in 77.8% of patients. Schüngel et al. (12) reported a permanent neurological morbidity rate of 4.8% at follow-up in 93% of patients, with an overall mortality rate of 2.3%. Hanel et al. (24) performed an analysis of these four studies of distal aneurysms treated with the SVB and reported a mortality rate of 2.5%, with three cases of neurological deaths (1.8%). Major stroke was observed in 1.2% of cases, and branch occlusion or stent thrombosis was observed in 5.5%.

When we compared the data obtained by Hanel et al. (24) with our case series, we did not find any major ischemic lesions, and the mortality rate was 0%. No cases of device thrombosis were reported; three patients presented with occlusion of the branches covered by the device, two of them in the perforator territory with delayed clinical presentation and favorable evolution (mRS score of less than 2 at the 3 months follow-up). Table 2 summarizes essential aspects of mortality, ischemic events, and stent thrombosis or branch occlusion comparatively.

**Table 2:** Comparison of studies of cerebral aneurysms treated with the SVB.

Authors	No. patients/aneurysms	Procedural complications	Major stroke	Permanent neurological deficit	Total mortality	Neurologic mortality	Stent thrombosis/branch occlusion
Bhogal et al. (9)	60/61	1.7%	1.6% (1/60)	1.07% (at 7.5 ± 4.2 months)	5.0% (3/60) (at 7.5 ± 4.2 months)	3.3% (2/60) (at 7.5 ± 4.2 months)	1.6% (1/60)
Gavrilovic et al. (10)	18/22	0%	0.0% (0/18)	22.2% (at 6 months)	0.0% (0/18) (at 6 months)	0.0% (0/22) (at 6 months)	9.1% (2/22)
Martínez-Galdámez et al. (11)	41/43	12.2%	0.0% (0/41)	0% (at 0 months)	0% (0/41) (at 0 months)	0% (0/41) (at 0 months)	0.0% (0/41)
Schüngel et al. (12)	44/47	15.9%	2.3% (1/44)	4.5% (at 7.7 months)	2.3% (1/44) (at 7.7 months)	2.3% (1/44) (at 7.7 months)	9.1% (4/44)
Hanel et al. (24)	163/173	NR	1.2% (2/163)	NR	2.5% (4/163) (at 6 months)	1.8% (3/163) (at 6 months)	5.5% (9/163)
Our series	50/50	8%	0% (0/50)	6% (at 12 months)	0.0% (0/50)	0.0% (0/50) (at 12 months)	6% (3/50)

SVB, Silk Vista Baby; NR, not reported.

### 4.3 Efficacy

The efficacy of endovascular treatment of distal cerebral aneurysms with the SVB was moderate in the early follow-up at 3 months, with an adequate occlusion rate of 56%. In the 6 months follow-up, an adequate occlusion rate of 81.6% was reported; in the last follow-up at 12 months, an adequate occlusion rate of 94% was reported.

When we compared our results with previous experiences using of the SVB (Table 3), we found that there were no significant differences concerning the degree of occlusion obtained. In their systematic review of 163 patients with 173 intracranial aneurysms, Hanel et al. (24) reported a complete or almost complete occlusion rate of 72.1% over the short-term follow-up.

**Table 3:** Angiographic results of the different studies that used the SVB.

Authors	No. patients/aneurysm	Follow-up (months)	Scale	Occlusion (*n*/*N*)
Bhogal et al. (9)	60/61	7.5 ± 4.2	RROC	Class I: 57.1% (32/56) Class II: 12.5% (7/56) Class III: 30.4% (17/56)
Gavrilovic et al. (10)	18/22	6	RROC	Class I: 76.9% (10/13) Class II: 15.4% (2/13) Class III: 7.7% (1/13)
Martínez-Galdámez et al. (11)	41/43	0	OKM	A: 60.5% (26/43) B: 9.3% (4/43) C: 11.6% (5/43) D: 18.6% (8/43)
Schüngel et al. (12)	44/47	7.7	OKM	A: 13.3% (4/30) B: 16.7% (5/30) C: 10.0% (3/30) D: 60.0% (18/30)
Hanel et al. (24)	163/173	6	RROC/OKM	(RROC-I/OKM D): 60.6% (60/99) (RROC-2/OKM C): 12.1% (12/99) Incomplete/not occluded: 27.3% (27/99)
Our Series	50/50	12	OKM	A: 2% (1/50) B: 4% (2/50) C: 28% (14/50) D: 66% (33/50)

SVB, Silk Vista Baby; no., number; OKM, O’Kelly–Marotta; RROC, Raymond–Roy.

Our study has a lower OKM D occlusion rate compared with previous studies that analyzed FDs in the proximal circulation. In the PUFS trial, Becske et al. (25) published initial results in 2013 and showed an aneurysmal occlusion rate of 73.6% at 6 months with progressive improvement at the 5 years follow-up (95.2%). Additionally, in the PEDESTRIAN study, Lylyk et al. (28) reported a complete occlusion rate of 75.8% at the 12 months follow-up. We consider that the lower rate of complete occlusion evidenced in our study is due to the presence of branches originating in the aneurysm itself, establishing, in these cases, a phenomenon of hemodynamic compensation that interferes with aneurysm closure and increases the number of cases classified as OKM C occlusion at the 12 months follow-up. In our series, of the 36 aneurysms with saccular morphology, 64% had at least one branch originating at the level of the sac or aneurysmal neck, 60% had complex aneurysms, and 25% had a dissecting fusiform aneurysm.

## 5 Limitations

The main limitation is that this study had a non-randomized design. Although this series is the largest reported to date, the small sample size limited subgroup analysis and the ability to identify differences in statistical comparisons where conceivable differences may exist.

Although the results showed an acceptable degree of efficacy and safety, more extensive, prospective, randomized studies with longer-term follow-ups are needed to corroborate the effectiveness of this treatment method and its comparison to other low-profile flow diverters.

In conclusion, treating complex intracranial aneurysms with the SVB was safe and effective. Long-term results showed high rates of adequate and stable occlusions.

## Data availability statement

The raw data supporting the conclusions of this article will be made available by the authors, without undue reservation.

## Ethics statement

The studies involving humans were approved by Comité de Etica Research Ethics Committee of the University Hospital Fundación Jiménez Díaz. The studies were conducted in accordance with the local legislation and institutional requirements. The participants provided their written informed consent to participate in this study.

## Author contributions

CR-F: Conceptualization, Data curation, Formal analysis, Funding acquisition, Investigation, Methodology, Project administration, Resources, Software, Supervision, Validation, Visualization, Writing – original draft, Writing – review & editing. PR-G: Conceptualization, Data curation, Formal analysis, Funding acquisition, Investigation, Methodology, Project administration, Resources, Software, Supervision, Validation, Visualization, Writing – review & editing. MG-S: Writing – review & editing. MM-Z: Conceptualization, Data curation, Formal analysis, Funding acquisition, Investigation, Methodology, Project administration, Resources, Software, Supervision, Validation, Visualization, Writing – review & editing. CT-I: Conceptualization, Data curation, Formal analysis, Funding acquisition, Investigation, Methodology, Project administration, Resources, Software, Supervision, Validation, Visualization, Writing – original draft. JE: Conceptualization, Data curation, Formal analysis, Funding acquisition, Investigation, Methodology, Project administration, Resources, Software, Supervision, Validation, Visualization, Writing – review & editing. MV: Conceptualization, Data curation, Formal analysis, Funding acquisition, Investigation, Methodology, Project administration, Resources, Software, Supervision, Validation, Visualization, Writing – original draft. LR: Conceptualization, Data curation, Formal analysis, Funding acquisition, Investigation, Methodology, Project administration, Resources, Software, Supervision, Validation, Visualization, Writing – original draft. AL: Conceptualization, Data curation, Formal analysis, Funding acquisition, Investigation, Methodology, Project administration, Resources, Software, Supervision, Validation, Visualization, Writing – review & editing. JP: Conceptualization, Investigation, Writing – original draft, Writing – review & editing.
